# Fabrication and Operation of an Oxygen Insert for Adherent Cellular Cultures 

**DOI:** 10.3791/1695

**Published:** 2010-01-06

**Authors:** Shawn Oppegard, Elly Sinkala, David Eddington

**Affiliations:** Bioengineering, University of Illinois at Chicago

## Abstract

Oxygen is a key modulator of many cellular pathways, but current devices permitting *in vitro* oxygen modulation fail to meet the needs of biomedical research.  The hypoxic chamber offers a simple system to control oxygenation in standard culture vessels, but lacks precise temporal and spatial control over the oxygen concentration at the cell surface, preventing its application in studying a variety of physiological phenomena.  Other systems have improved upon the hypoxic chamber, but require specialized knowledge and equipment for their operation, making them intimidating for the average researcher.  A microfabricated insert for multiwell plates has been developed to more effectively control the temporal and spatial oxygen concentration to better model physiological phenomena found *in vivo* . The platform consists of a polydimethylsiloxane insert that nests into a standard multiwell plate and serves as a passive microfluidic gas network with a gas-permeable membrane aimed to modulate oxygen delivery to adherent cells.  The device is simple to use and is connected to gas cylinders that provide the pressure to introduce the desired oxygen concentration into the platform.  Fabrication involves a combination of standard SU-8 photolithography, replica molding, and defined PDMS spinning on a silicon wafer.  The components of the device are bonded after surface treatment using a hand-held plasma system.  Validation is accomplished with a planar fluorescent oxygen sensor.  Equilibration time is on the order of minutes and a wide variety of oxygen profiles can be attained based on the device design, such as the cyclic profile achieved in this study, and even oxygen gradients to mimic those found *in vivo* .  The device can be sterilized for cell culture using common methods without loss of function.  The device's applicability to studying the *in vitro* wound healing response will be demonstrated.

**Figure:**
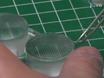


## Protocol

### 1. Device function

The hypoxic insert device contains 6-pillars that nest into a standard 6-well plate.  Gas flows into the pillar, across a microfluidic network at the base of the pillar, and flows back out of the device.  At the base of the pillar forming the bottom wall of the microchannel is a 100 μm thick gas-permeable PDMS membrane that permits oxygen diffusion between the gas microchannel and the culture media.  Thus, the device operates by establishing a concentration gradient that drives the media oxygen concentration towards a desired value.
The device offers a number of advantages over the hypoxic chamber and other cell oxygenation systems: 1) minimizes the oxygen diffusion path permitting faster temporal control, 2) enhances the spatial control over oxygenation, as dependent on the pillar microchannel design, 3) possesses a smaller lab footprint and higher throughput to increase experimental efficiency, and 4) adapts to common cell culture tools (e.g. multiwell plate) without the requirement for specialized knowledge and equipment.

### 2. Device fabrication

First the pillar array is replica molded with PDMS in a previously machined Delran mold.
Next the gas microchannel at the bottom of each pillar is fabricated using standard SU-8 photolithography and PDMS replica molding.
The gas-permeable membrane is fabricated by defined spinning of PDMS on a silicon wafer to achieve the desired thickness.  In this example we use a 100 μm thick membrane which was fabricated by spinning 500 rpm for 10 seconds and then 900 rpm for 30 seconds.
All components are bonded together after oxygen plasma treatment with a handheld plasma device (Model BD-20, Electro-Technic Products).

### 3. Device setup

Gently insert device into the plate, making sure to avoid bubbles.  Angling the device during insertion helps to expel bubbles out one side.  For actual cell-based experiments, this step must be done inside a sterile laminar flow hood.  The chances for contamination are greatly reduced once the device is inserted, so the assembly can be carried over to the incubator in a non-sterile environment.
Connect tubing from the source gas tank to the inlet and outlet ports of the device.  For cell-based experiments, the culture incubator should have a hole to permit tubing entry and the tubing should be connected after carrying the device and placing it into the incubator.  Be careful to avoid putting excess pressure on the device, which could deform the PDMS enough to crush the underlying cells.
Make sure the precision flow regulator is closed before opening the tank top regulator to avoid overflowing the device.  Start the flow of gas.  Gently open precision flow regulator to desired flow rate (50-100 mL/min).  Watch the value closely over the next hour and make adjustments as needed, since the pressure drop will change while the system equilibrates, altering the flow rate.  To avoid formation of bubbles in the media, reduce the flow rate to between 10-20 mL/min after an initial 15 minute equilibration duration with the higher flow rate.
After desired experimental duration, stop gas flow, remove the plate, and process cells accordingly (e.g. lyse, stain, count, etc.).

### 4. Device Validation

**Calibration**
  Select the number and location of the positions on the fluorescent oxygen (FOXY) sensor slide (depending on the validation requirements) that will be used for oxygen measurement.  The slide contains a fluorescent ruthenium dye coating that is quenched by oxygen.
  Expose slide directly to 0, 10, and 21% oxygen from gas tank and capture images after 5 minutes for proper equilibration.
  Export the mean image intensity for each position and plot oxygenation concentration as dependent on fluorescent intensity.
  Generate calibration curve by fitting linear curves to the 0-10% line and 10-21% line.
**Heterogeneity**
  Establish multiple points spanning the width of the channel at a defined interval (e.g. every 1 mm).  Note that there may be overlap of images depending on the spacing.
  Measure the oxygen concentration at the well surface through the device
**Equilibration**
  Choose three points on the FOXY slide in which to measure the oxygen concentration.
  Immediately after capturing a first image at ambient oxygen levels, open the precision flow regulator to start gas flow into the device.  Capture images at an appropriate interval to assess the duration and extent of oxygen concentration equilibration (e.g. every 10 seconds for 30 min).

### 5. Applications

**Wound Healing**
  A day before the experiment, soak the sterilized PDMS insert in serum-free medium to reduce the inhibit gas bubble formation in the well.
  Culture cells to 100% confluency in a 6-well plate.
  Create straight scratches in the monolayers with a p200 pipette tip to simulate wounds.
  Aspirate the cell media, rinse with 5 mL media, and aspirate again. It is important to not disturb the monolayer of cells.
  Refill the wells with 4 mL serum-free medium to reduce cell proliferation.
  Place insert into the wells and connect each well to a corresponding oxygen concentration.
  Place 6-well plate with insert on heated stage at 37°C.
  Capture time lapse images of the cells at the desired interval and total duration. MATLAB's *T_Scratch* , a wound measuring algorithm, can be used to analyze the unhealed surface area.

### 6. Representative Results

**Device validation** The hypoxic insert device exhibits great improvements over the hypoxic chamber in terms of oxygen equilibration time and extent, requiring less than 2 minutes to stabilize to 0.5% oxygen. The device membrane-well bottom gap size was the critical factor in determining equilibration efficiency, with larger gap sizes requiring more time to reach steady-state oxygen concentration values. The device also permits a great deal of control over the spatial oxygenation in a single well, allowing the formation of multiple conditions, and even generating a cyclic oxygen profile across the surface of the well.
**Wound healing** Cell monolayers were exposed to 10% or 21% oxygen and the surface area of the wound was analyzed over time.  Scratches exposed to 21% oxygen closed the slowest and 10% the fastest.  Figure 1 shows images of a scratch over the course of 17 hours.  The graphs in Figure 5 show the percent of open wound area for both oxygen concentrations for the duration of the experiment.

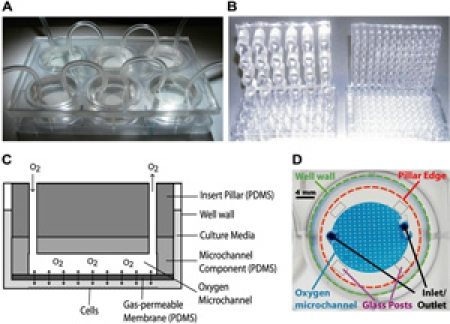
 Click here to see a larger figure. **Figure 1.** Schematic and diagrams illustrating device features.  The oxygen insert device is fabricated by conventional photolithography (microfluidic network), replica molding (microfluidic network and insert scaffold), and defined spinning of PDMS (gas-permeable membrane). A) The oxygen device nested into a 6-well plate.  B) Examples of 24 and 96-well pillar arrays.  C) A cross-sectional schematic of a pillar.  Oxygen flows into the device through the inlet and travels across a microfluidic network at the bottom of the pillar.  Oxygen can freely diffuse across the gas-permeable PDMS membrane at the bottom of the pillar and dissolve into the culture media.  D) A microscope image showing the various features of a single-channel pillar from above, with bonded glass posts for the equilibration studies.


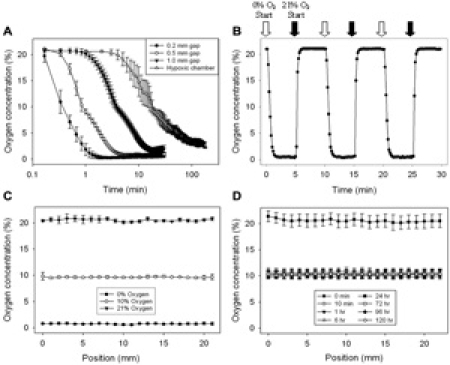
 Click here to see a larger figure. **Figure 2.** Validation of the device with oxygen sensors. Oxygen tension within each well was characterized using a planar ruthenium oxygen sensor.  All oxygen mixtures contained balanced nitrogen and 5% CO_2_ for media buffering.  A) Plot illustrating the effect of post height, and thus oxygen diffusion distance between the membrane and cells, on the equilibration time and effectiveness.  Heights were established by cut-glass posts bound to the bottom of the device.  All three post sizes yield equilibration times much improved over the hypoxic chamber.  Note that time is on a log scale.  B) Plot depicting the rapid oxygen equilibration response time of the 0.2 mm gap device.  C) Multi-position linescans were also taken across the well under the microchannel to ensure homogeneity of the oxygen concentration introduced by the device.  Graph depicts the oxygen concentration measured after infusing 0%, 10%, and 21% oxygen for 10 min.    D)  Device effectively maintains 10% oxygen over 5 days.


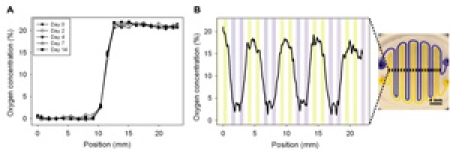
 Click here to see a larger figure. **Figure 3.** Experimentation with more complex oxygen microchannel designs. A) Dual-condition microchannel setup yields a stable 0% and 21% oxygen profile over 14 days. B) An interdigitated and winding pattern of 500 μm width microchannels extending across the pillar results in a cyclic oxygen profile.  Note that the data only depicts one representative trial as microchannel alignment was difficult.


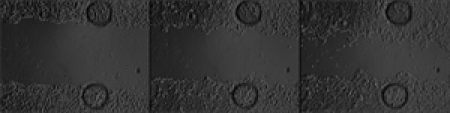
 Click here to see a larger figure. **Figure 4.** Timelapse images of wound closure 0, 7, and 17 hours after initial scratch. Cells were delivered 21% oxygen throughout duration of experiment.


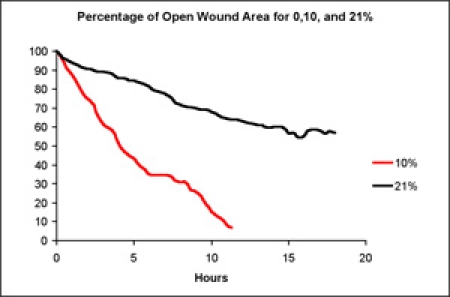
 Click here to see a larger figure. **Figure 5.** Effect of oxygen concentration on wound healing rate in a scratch assay.

## Discussion

The device is fabricated by standard SU-8 photolithography, replica molding, and defined spinning and made entirely of polydimethylsiloxane.  Gas is introduced into the device to establish a concentration gradient between the pillar microchannel and the culture media, driving the system towards a desired equilibrium oxygen concentration.  The device has been shown to effectively modulate the temporal and spatial oxygenation inside a well, as well as modulate cellular behavior appropriately.  The spatial patterning of oxygenation is defined by the microchannel at the base of the pillar, so a variety of designs could be implemented in crafting the photomask.  Additionally, infusion of the desired gas into the gas-phase of the well is expected to improve equilibration time and extent of hypoxia.  A microfluidic mixing network could be adapted to the device to provide a means to produce novel gas mixtures from only a few stock gas tanks.  Finally, a mechanism for media exchange would eliminate the need for removal of the device from the multiwell plate, of which the cells may respond.

The device has applications in any *in vitro* or *ex vivo* experiment requiring control over oxygen concentration.  As oxygen is an important physiological variable affecting a vast majority of signaling pathways, the areas of research that would benefit is limited by the creativity of the researcher.  Some fields that would benefit from the enhanced temporal control of oxygen concentration include cancer metastasis, sleep apnea, and cardiac ischemia reperfusion injury, among many others.  For example, intermittent hypoxia has been correlated with more invasive cancers, upregulating a number of metastastis-associated genes relative to continuous hypoxia and normoxia.  Spatial control is also important, as oxygen gradients are critical in development, liver zonation, drug toxicity, and the stem cell niche. The device presented in this article will benefit a number of areas of research by providing a system with a smaller lab footprint, relatively simple operational requirements, and far greater control over oxygen exposure to cells.

